# Insights into transcriptional expression and putative functions of multiple polyhydroxyalkanoate synthase paralogs in *Haloferax mediterranei*

**DOI:** 10.1093/femsle/fnag006

**Published:** 2026-01-19

**Authors:** Chloé Vanden Haute, Brendan Schroyen, Ulrich Hennecke, Eveline Peeters

**Affiliations:** Research Group of Microbiology, Department of Bioengineering Sciences, Vrije Universiteit Brussel, Pleinlaan 2, B-1050 Brussels, Belgium; Research Group of Microbiology, Department of Bioengineering Sciences, Vrije Universiteit Brussel, Pleinlaan 2, B-1050 Brussels, Belgium; Research Group of Organic Chemistry, Department of Chemistry, Vrije Universiteit Brussel, Pleinlaan 2, B-1050 Brussels, Belgium; Research Group of Microbiology, Department of Bioengineering Sciences, Vrije Universiteit Brussel, Pleinlaan 2, B-1050 Brussels, Belgium

**Keywords:** *Halobacteriales*, polyhydroxyalkanoates, bioinformatics, paralogs, RT-qPCR, archaea

## Abstract

The halophilic archaeon *Haloferax mediterranei* is a promising candidate for polyhydroxyalkanoate (PHA) production, offering several advantages due to its extremophilic physiology. While its primary PHA synthase, a class III enzyme composed of PhaC_Hme_ and PhaE_Hme_ subunits, has been well characterized, the genome encodes three additional *phaC* paralogs (*phaC_1_, phaC_2_*, and *phaC_3_*), which were previously labeled as cryptic and remain poorly understood. In this study, we systematically investigated these paralogs by employing a targeted bioinformatics pipeline, revealing notable diversity in PHA synthases among *Halobacteriales* and underscoring the distinctiveness of *H. mediterranei*. We further analysed the native transcriptional expression profiles of all *phaC* paralogs under three physiologically relevant conditions: growth-limiting and growth-permissive conditions, as well as valeric acid supplementation to alter PHA monomer composition. Quantitative RT-PCR analysis demonstrated that all three paralogs are transcriptionally active and differentially expressed, refuting earlier assumptions of their cryptic nature. Expression patterns were found not to correlate to polymer composition but to be dependent on growth phase, suggesting a potential physiological role for each paralog in native PHA metabolism. These findings offer new insights into the functional complexity of PHA biosynthesis in *H. mediterranei* and lay the groundwork for future metabolic engineering aimed at optimizing biopolymer production.

## Teaser

*Haloferax mediterranei* exhibits distinct expression of multiple polyhydroxyalkanoate synthase paralogs, providing new insights into archaeal biopolymer metabolism and potential strategies for advancing sustainable polyhydroxyalkanoate production.

## Introduction

The search for sustainable alternatives to petrochemical polymers has directed growing interest toward bioplastics such as polyhydroxyalkanoates (PHAs). These biodegradable polyesters possess thermomechanical properties comparable to polypropylene and polyethylene (Laycock et al. 2013) and are produced by microbial biosynthesis. Beyond serving as carbon and energy storage (Dawes and Senior 1973), PHAs can enhance host tolerance to environmental stress, such as ultraviolet irradiation, desiccation, and osmotic stress (Tal and Okon 1985), although the underlying mechanisms remain incompletely understood (Obruca et al. 2018).

Despite the potential, microbial PHA production remains several times more expensive than petrochemical counterparts (Tan et al. 2021). Extremophilic hosts have been proposed as a strategy to improve cost efficiency, offering advantages in fermentation and downstream processing (Kalia et al. 2025). Among them, the extremely halophilic archaeon *Haloferax mediterranei* has emerged as a promising candidate: its use as a production host can reduce costs by over 20% (Chen et al. 2022) through reduced sterility requirements and simplified cell lysis by hypoosmotic shock in fresh water (Chen et al. 2006, Hermann-Krauss et al. 2013). As an archaeon, it also produces endotoxin-free PHAs, an advantage over bacterial hosts such as *Cupriavidus necator* in biomedical applications (Koller et al. 2013). Moreover, *H. mediterranei* grows on diverse low-cost carbon sources including dairy whey, glycerol from the biodiesel industry, vinasse and stillage from the bioethanol industry, chitin and crop residues (Koller et al. 2007a, Bhattacharyya et al. 2012, 2014, Hermann-Krauss et al. 2013, Hou et al. 2013, Alsafadi et al. 2020).

A notable feature of *H. mediterranei* is its natural ability to synthesize poly(3-hydroxybutyrate-co-3-hydroxyvalerate) (PHBV) (Don et al. 2006). PHBV offers superior material properties compared to the more common poly(3-hydroxybutyrate) homopolymer, being less crystalline, more flexible, and having a larger processing window (Luzier 1992). Unlike most production hosts, which require the addition of costly precursor molecules to synthesize PHBV, *H. mediterranei* synthesizes the copolymer directly from unrelated carbon sources (Han et al. 2013).

The key enzyme in the PHA synthesis pathway of *H. mediterranei* is a class III PHA synthase consisting of PhaE_Hme_ and PhaC_Hme_ (Lu et al. 2008). Genome sequencing has revealed three additional *phaC* paralogs: *phaC_1_, phaC_2_*, and *phaC_3_* (Han et al. 2010). Under previously investigated PHA-accumulating conditions, none of the three paralogous *phaC* genes were transcribed and they were thus labeled as cryptic (Han et al. 2010), consistent with the observation that deletion of *phaC_Hme_* completely abolished PHA synthesis (Lu et al. 2008). However, heterologous expression studies in *Haloarcula hispanica* indicated that PhaC_1_ and PhaC_3_, but not PhaC_2_, can form functional complexes with PhaE_Hme_, producing polymers with varying 3-hydroxyvalerate (3HV) content (Han et al. 2010). Furthermore, deletion of a gene encoding an unusual phosphoenolpyruvate synthetase-like protein and located in a divergent operon with *phaC_1_*, induced the expression of *phaC_1_* and *phaC_3_*, leading to the synthesis of PHA polymers with distinct monomeric compositions (Chen et al. 2020). These findings suggest that the paralogs could potentially be exploited for generating tailor-made copolymers in *H. mediterranei*.

Despite the presence of four *phaC* paralogs, of which minimally three have a functional capability, the extent to which cultivation conditions influence their transcription remains unclear, and a comparative structure–function analysis has not yet been performed. In this study, we investigate the role of PhaC_Hme_ and its paralogs in native PHA biosynthesis of *H. mediterranei*. By characterizing transcriptional expression profiles and the chemical composition of PHBV polymers in different growth conditions and phases, as well as modeling structural features of the corresponding enzymes, we aim to contribute to fundamental insights to support the rational design of strategies for production of customized PHAs.

## Materials and methods

### Bioinformatic analysis

Genomic neighborhoods were analysed using the accession numbers and genome assembly provided by the National Center for Biotechnology Information (NCBI). Protein structures were modeled using AlphaFold 3 (Abramson et al. 2024). Multiple sequence alignments were generated with COBALT (Papadopoulos and Agarwala 2007).

To develop the bioinformatics pipeline, all *H. mediterranei* PhaC proteins (WP_004056138.1, WP_004060157.1, WP_004061055.1, and WP_004060696.1) and the associated PhaE_Hme_ subunit (WP_004056139.1) were retrieved from the NCBI RefSeq database and used as reference queries. A dataset of PhaC-containing target species within the order *Halobacteriales* was compiled by extracting unique scientific names from an initial set of BLAST hits. Proteomes were downloaded using the NCBI Entrez API and concatenated to generate a single reference database representing *Halobacteriales*. A local BLAST database was constructed using the makeblastdb utility from BLAST+ (v2.16.0). Protein–protein BLAST (BLASTp) was run using each of the four PhaC reference sequences against the custom database, with the top four hits retained per query (-max_target_seqs 4) (Camacho et al. 2009). Output was formatted with extended fields (qacc sacc pident length mismatch gapopen qstart qend sstart send evalue bitscore stitle) to allow in-depth postprocessing. A separate BLASTp search was conducted with PhaE_Hme_ (WP_004056139.1) as the query against the same database to assess genomic colocalization of PhaC and PhaE. BLAST results were parsed in Python (v 3.11) using pandas. Duplicate hits were removed based on identical alignment statistics across multiple fields and for each reference class (PhaC_Hme_, PhaC_1_, PhaC_2_, and PhaC_3_), the highest scoring unique hit per organism was retained. The identity of the best matching reference for each hit was inferred by selecting the class with the highest % identity. A hit was defined as weak when the % identity of the best match was below 65%. To examine truncation patterns, the alignment length was compared to the full length of the reference sequence, expressed as a percentage of query coverage. To determine the genomic context of the genes corresponding to each PhaC and PhaE hit, genomic coordinates were retrieved using the NCBI Entrez efetch API. These coordinates were used to identify the closest PhaC-encoding gene for each hit encoding PhaE within the same genome, based on the smallest absolute distance between the end coordinate of *phaE* and the start of the corresponding *phaC* gene. This information was used to infer colocalized gene pairs and assign each PhaE to its most likely functional PhaC partner.

Strong hits (% identity ≥ 65%) were classified as either PhaC_Hme_-, PhaC_1_-, PhaC_2_-, or PhaC_3_-like based on identity scores and visualized in Python (v 3.11) using matplotlib. To assess patterns of paralog occurrence, summary statistics were calculated from the final dataset. Species with one or more PhaC paralogs were tallied, and the most prevalent number of paralogs was identified. The distribution of best-matching classes was examined. Additionally, the number of species for which a PhaC–PhaE pairing could be confidently established based on genomic colocalization was quantified. Each strong PhaC hit was represented as a colored circle according to its best matching class, plotted per organism and vertically stacked.

### Strains, media and cultivation conditions

*Haloferax mediterranei* DSM1411 was cultivated at 42°C and 125 rpm in an orbital shaker in different liquid defined media derived from the Hv-min minimal medium (Dyall-Smith 2009). Briefly, this medium contained 15.9 mM Tris–HCl (pH 7.5), 2.6 M NaCl, 93.8 mM MgCl_2_, 90.3 mM MgSO_4_, 60 mM KCl, 5.3 mM NH_4_Cl, 6.4 mM CaCl_2_, 0.6 mM K_2_HPO_4_, 0.4 mM KH_2_PO_4_, 1.9 µM MnCl_2_, 1.6 µM ZnSO_4_, 8.8 µM FeSO_4_, 0.2 µM CuSO_4_, 3.2 µM thiamine, and 0.4 µM biotin. This medium was supplemented either with 32.0 mM sodium dl-lactate, 3.7 mM glycerol and 15.0 mM sodium succinate dibasic hexahydrate (labeled “Hv-min” with a C/N/P ratio of 62.8:2.3:1.0) or only with 74.2 mM glycerol (labeled “glycerol medium” with a C/N/P ratio of 83.6:2.3:1.0) as carbon source. Growth was monitored by measuring optical density at 600 nm (OD_600_). When mentioned, the Hv-min medium was supplemented with 0.5 g l^−1^ valeric acid in exponential phase (OD_600_ between 0.4 and 0.6) (C/N/P ratio of 71.9:2.3:1.0).

### Nucleic acid extractions and cDNA preparation

For genomic DNA (gDNA) extraction, *H. mediterranei* cells were harvested by centrifugation at 16 000 × *g* during 2 min followed by resuspension in Elution Buffer (“EB” 10 mM Tris–HCl pH 8.5) and addition of an equal volume of phenol/chloroform/isoamyl alcohol solution (25:24:1). This solution was vortexed, centrifuged at 16 000 × *g* during 5 min, the top aqueous phase was recovered and this procedure was repeated. Next, an equal volume of chloroform/isoamyl alcohol solution (24:1) was added and the top aqueous phase was removed in the same manner. This phase was combined with 0.75 M NH_4_OAc and 20 µg glycogen, followed by ethanol precipitation after which the DNA-containing pellet was resuspended in EB.

RNA was extracted using a RNeasy Mini Kit (Qiagen) with two consecutive DNase treatments according to manufacturer’s instructions. The starting culture volumes for extractions at different growth phases were normalized to the OD_600_, in order to start from similar amounts of cells. RNA was converted to cDNA using a GoScript^TM^ Reverse Transcriptase kit (Promega) following manufacturer’s instructions.

### Gene expression analysis by quantitative RT-PCR

Specific primers for quantitative RT-PCR (RT-qPCR) were designed employing Primer3 (Untergasser et al. 2012) (Table S1). For each primer pair, amplification efficiency and primer specificity was verified employing a 10-fold dilution series of gDNA as a template. RT-qPCR reactions were performed with GoTaq qPCR Master Mix (Promega) and 10 µM of each primer in a CFX96 Real-Time System (Bio-Rad). Each condition was analysed using three biological replicates in two technical replications. The following thermal cycling program was performed: 3 min at 95°C followed by 40 cycles of 10 s at 95°C and 30 s at 55°C.

The Pfaffl method was used to calculate relative gene expression ratios (Pfaffl 2001). As a housekeeping gene, either *tbp* (E6P09_03900) or *ffs* (E6P09_08165) was used, or the geometric means of both (Vandesompele et al. 2002; Supplementary Note S1). Statistical analysis was performed by averaging technical replicates and comparing the resulting groups of three biological replicates by means of an unpaired Student’s *t*-test in the Prism 10 software package (GraphPad). Normality of the data was verified using a Shapiro–Wilk test in the Prism 10 software package (GraphPad).

Quantitative transcript levels were estimated by relating the measured Ct values of RT-qPCR assays to the standard curves recorded for each gene (Fig. S2 and Table S2). The DNA quantities were then used as a proxy for transcript levels, allowing the comparison of different paralogs at different timepoints.

### PHA extraction

*Haloferax mediterranei* cells were harvested in stationary phase (OD_600_ around 2.0 for Hv-min, 2.7 for Hv-min with valeric acid, and around 0.5 for glycerol medium), centrifugated at 12 100 × *g* during 15 min, washed with HSPB buffer (2 M NaCl, 71.7 mM K_2_HPO_4_, and 28.3 mM KH_2_PO_4_) and snap-frozen in liquid nitrogen. When frozen, the cells were lyophilized for 16 h in a benchtop lyophilizer (Labconco). The dried pellets were weighed and placed in a Whatmann Extraction Thimble, which was suspended in 30 ml ethanol and incubated at room temperature while shaking for 16 h. The thimble was transferred to 30 ml chloroform and incubated. Lastly, chloroform was allowed to evaporate overnight, after which the resulting PHA was weighed.

### Nuclear magnetic resonance analysis

The monomeric composition of the extracted PHA polymers was determined by proton nuclear magnetic resonance (^1^H-NMR) spectroscopy. Polymers were dissolved in deuterated chloroform (CDCl_3_) and ^1^H-NMR spectra were measured at 400 MHz on a Bruker Avance Neo spectrometer (Bruker, Germany) using a standard pulse sequence at ambient temperature. The chemical shifts are reported in delta (δ) units in parts per million (ppm) relative to trimethylsilane. Spectra were referenced relative to the residual solvent signal. The 3HV content of the polymers was calculated from the ratio of the area under the methine proton peaks of the 3-hydroxybutyrate and 3HV subunits, respectively.

## Results

### Comparative analysis of PhaC paralogs in *H. mediterranei*

**Figure 1:**
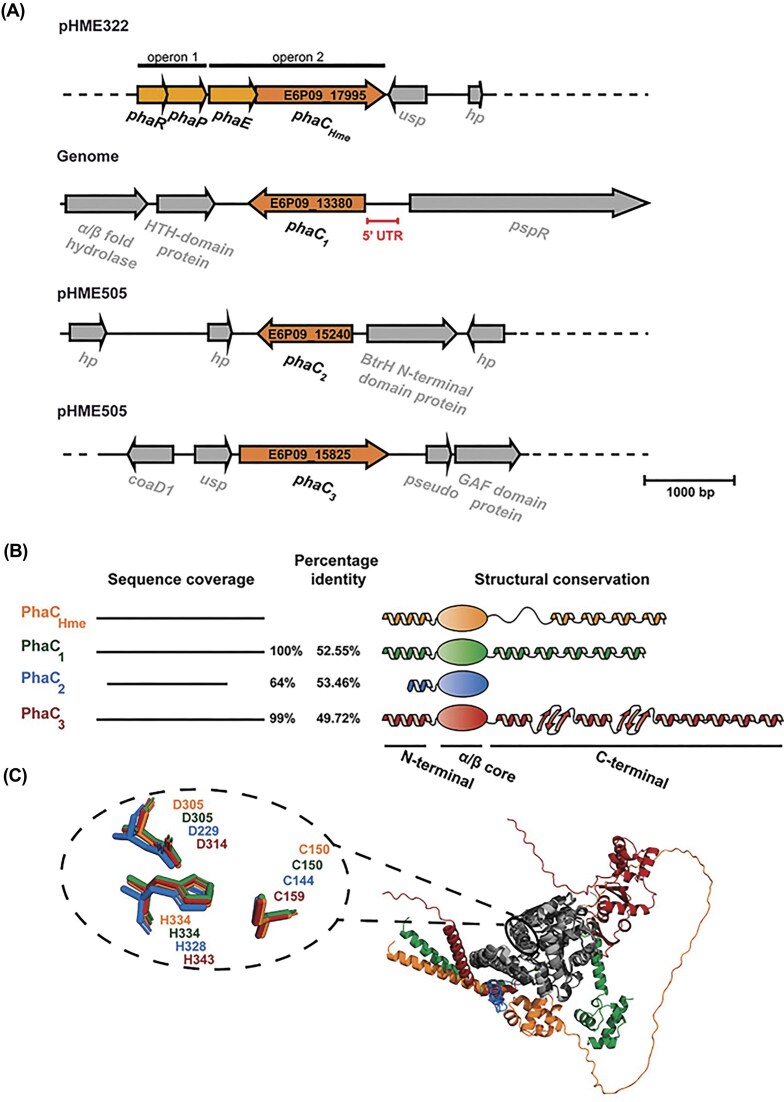
Genome organization of *phaC* paralogs in *H. mediterranei* and sequence and structural similarities of the resulting proteins. (A) Schematic overview of the genome organization of the four *phaC* paralogs in *H. mediterranei*. Usp: universal stress protein, hp: hypothetical protein, HTH: helix-turn-helix, *pspR*: PPS-like regulator, BtrH: Butirosin biosynthesis protein H, and *coaD1*: phosphopantetheine adenylyltransferase. (B) Sequence coverage with respect to the PhaC_Hme_ sequence, with indication of sequence identities of PhaC_1_, PhaC_2_, and PhaC_3_ with respect to PhaC_Hme_. On the right, a schematic representation is shown of the domain organization of the PhaC enzymes, with emphasis on their secondary structure elements. (C) Structural superposition of the catalytic cores of the four enzymes, with a zoomed view of the three catalytically indispensable residues and their respective positions within the PhaC structures.

All four *phaC* paralogous genes are located at distinct genomic loci in *H. mediterranei*, including the chromosome and extrachromosomal elements (Fig. 1A). While *phaC_Hme_*, which is considered to encode the main PhaC enzyme in *H. mediterranei*, is located on the pHM322 megaplasmid in an operon with the gene encoding its auxiliary protein PhaE_Hme_ (Lu et al. 2008) adjacent to another operon with other PHA-related genes, the other paralogs are encoded on genomic locations without any PHA-related genes in their neighborhood. Whereas *phaC_1_* is located on the chromosome, the *phaC_2_* and *phaC_3_* genes are encoded on the megaplasmid pHME505 (Fig. 1A).

Based on a multiple sequence alignment (Fig. S3), it can be observed that PhaC_1_ and PhaC_3_ cover nearly full sequence length of the primary enzyme PhaC_Hme_, while PhaC_2_ only covers 62% of the sequence, missing segments at the C- and N-termini (Fig. 1B). All paralogs exhibit between 44% and 60% sequence identity (Fig. S4). Structural modeling confirmed with high confidence that the conserved central segment of the proteins corresponds to the α/β core, which is a signature for the α/β hydrolase superfamily (Fig. 1; Fig. S5). Within this core, the indispensable catalytic triad, consisting of a histidine, cysteine, and aspartate residue, is positionally conserved (Fig. 1C). In addition the CAP domain, which functions to close the active site and to block the substrate entry path, is conserved as well (Chek et al. 2017). In contrast to the core of the enzyme, structural features of the termini are predicted with a lower confidence for all paralogs, in agreement with a lower conservation. While all paralogs are predicted to form an α helix at the N-terminus, albeit being much smaller for PhaC_2_, its relative position varies significantly (Fig. 1B). The N-terminal PhaC domain is hypothesized to be involved in different types of interactions including protein–granule interactions and the formation of dimers or other protein–protein interactions (Neoh et al. 2022). A large variability in terms of length, is observed for the C-terminal region.Whereas PhaC_2_ lacks a C-terminal domain, PhaC_3_ is predicted to form an extended C-terminal domain with two additional β sheets. The absence of this C-terminal region in PhaC_2_ might render the enzyme nonfunctional as previously shown (Han et al. 2010).

### Phylogenetic distribution of PhaC in *Halobacteriales*

**Figure 2:**
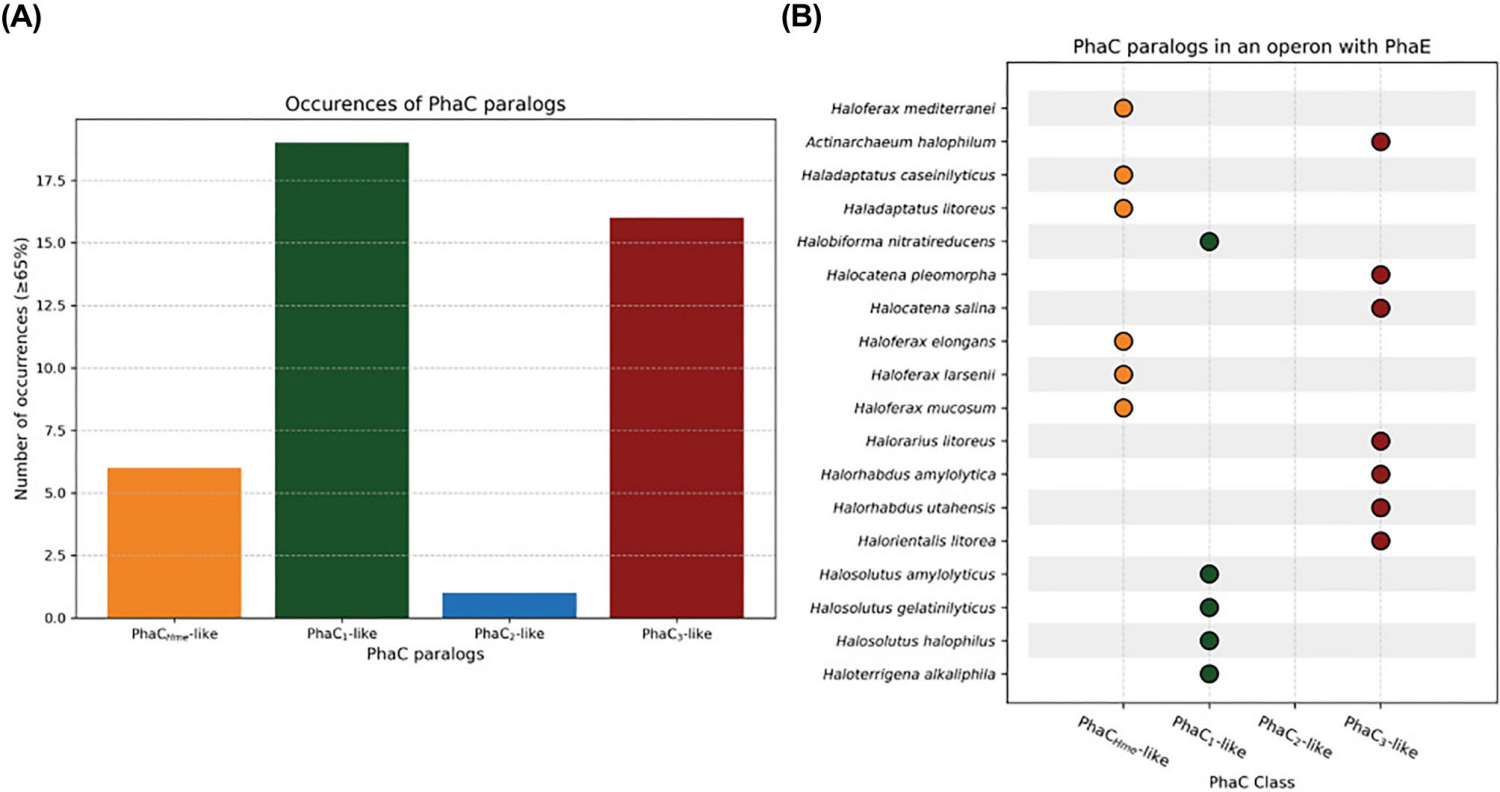
Graphical representation of the occurrence of PhaC paralogs in the genomes of PHA-producing *Halobacteriales*. PhaC homologs were classified into four categories—PhaC_Hme_-like, PhaC_1_-like, PhaC_2_-like, or PhaC_3_-like—based on sequence identity. (A) Bar chart representing the distribution of PhaC-like paralogs in *Halobacteriales*. (B) Overview of PhaC-like paralogs encoded in the vicinity of a PhaE subunit, suggesting the presence of a *phaEC* operon.

To determine whether the occurrence of multiple PhaC paralogs is a conserved trait in PHA-producing *Halobacteriales*, we screened genomes predicted to contain at least one identified *phaC* homolog that were thus considered putative PHA producers (Fig. 2). Of 225 genomes, only a limited number harbor more than one *phaC* gene: 32 species were predicted to encode two paralogs, four species to encode three, while *H. mediterranei* is the only species with four paralogs.

Upon comparing the detected PhaC sequences with each of the *H. mediterranei* paralogs, a scattered phylogenetic occurrence was observed, where the majority showed the highest sequence identity with either PhaC_1_ or PhaC_3_ of *H. mediterranei* (Fig. 2A). In contrast, C-terminal truncated PhaC_2_-like homologs were not detected in any other species, suggesting that this paralog may be a unique evolutionary feature in *H. mediterranei*. In addition to *phaC* diversity, patterns in *phaE* colocalization were examined (Fig. 2B). It was observed that the gene encoding the PhaE subunit frequently cooccurs in the genome with various *phaC* paralogs, not exclusively with one particular class. This indicates that these PhaC paralogs probably function in a similar way as PhaC_Hme_ in *H. mediterranei*. When a phylogenetic tree is constructed, homologs exhibiting more than 65% sequence identity cluster together with similar paralog-types and cluster together with the paralogs of *H. mediterranei* (Fig. S6). However, PhaC sequences within these clusters are often located outside of a PhaEC operon. This further proves evolutionary differentiation. Despite the presence of multiple *phaC* genes in some genomes, most species contained only a single copy of *phaE*. Only in a few exceptional cases, for example in *Natronomonas aquatica*, more than one *phaE* gene was identified.

### Transcriptional expression of *phaC* paralogs in different medium conditions

**Figure 3:**
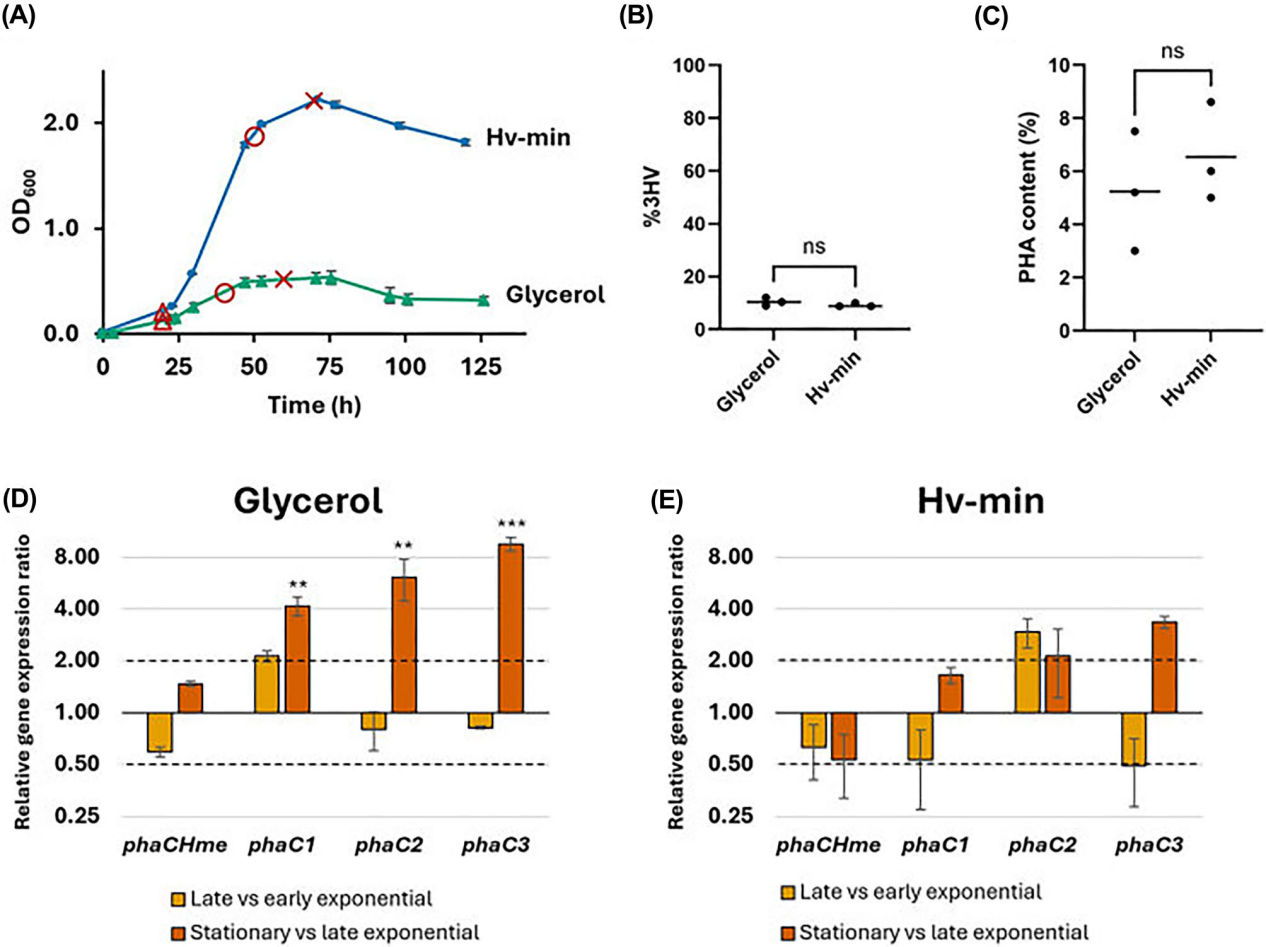
Growth, PHA synthesis and relative gene expression of *phaC* paralogs in growth-limiting and -permissive conditions (A) Growth curves of *H. mediterranei* in glycerol medium and Hv-min defined media, measured as optical density at a wavelength of 600 nm (OD_600_) over time. Values represent the average of three biological replicates and error bars indicate standard deviations. mRNA sampling points are indicated with triangles for early exponential, circles for late exponential, and crosses for stationary phase. (B) 3-Hydroxyvalerate (%3HV) content in the PHA polymers produced in glycerol and Hv-min media, as measured by NMR. Triplicate values were compared using a *t*-test; ns = not significant. (C) PHA content in glycerol and Hv-min media. Mean values are indicated with a horizontal line. Triplicate values were compared using a *t*-test; ns = not significant. (D) Relative transcriptional expression of *phaC* paralogs in growth-limiting conditions, as determined by RT-qPCR. Values represent the average of three biological replicates and error bars indicate standard deviations. Ct values were compared using a statistical *t*-test. ** indicates *P* = .01–.001, *** indicates *P* < .001. Dotted lines indicate thresholds for 2.0 (upregulation) and 0.5 (downregulation). (E) Relative transcriptional expression of *phaC* paralogs in growth-permissive conditions, as determined by RT-qPCR. Values represent the average of three biological replicates and error bars indicate standard deviations.

To investigate whether the four *phaC* paralogs are transcriptionally expressed and how their expression changes with different carbon sources, we selected two defined media: (i) Hv-min medium, containing a mixture of glycerol, sodium lactate, and sodium succinate and (ii) glycerol medium, containing only glycerol (Fig. 3). First, these media were compared for their ability to support growth and PHA production in *H. mediterranei*. While the glycerol medium supported growth to a maximal OD_600_ of 0.533 ± 0.062, which is relatively low for this species, the Hv-min medium reached a much higher carrying capacity with a maximal OD_600_ of 2.220 ± 0.003 (Fig. 3A). It can thus be concluded that glycerol medium represents a growth-limiting condition and Hv-min medium a growth-permissive condition. In terms of PHA production, both media performed similarly. Nuclear magnetic resonance (NMR)analysis (Figs S7 and S8) demonstrated that the PHBV composition was comparable in both growth conditions with 10.4 ± 1.3%3HV and 9.2 ± 0.6%3HV in the glycerol and Hv-min medium, respectively (Fig. 3B). The PHA content was relatively low, namely 5.2 ± 1.8% in glycerol medium and 6.5 ± 1.5% in Hv-min medium (Fig. 3C). Despite large differences in biomass yield, both media supported similar PHA production, suggesting that PHA synthesis in *H. mediterranei* is largely independent of growth rate. These two media therefore provide suitable conditions to investigate *phaC* expression and its regulation in a comparative manner, as PHA synthesis is relevant in both conditions, yet the cells are in markedly different metabolic and physiological states.

In both the growth-limiting (glycerol medium) and growth-permissive (Hv-min medium) condition, transcriptional expression levels of *phaC_Hme_, phaC_1_, phaC_2_*, and *phaC_3_* were monitored employing RT-qPCR (Fig. 3D and E). In contrast to previous claims that *phaC_1_, phaC_2_*, and *phaC_3_* are cryptic genes (Han et al. 2010, Chen et al. 2020), we observed transcriptional activity for all *phaC* paralogs in these conditions. Moreover, in the growth-limiting condition, *phaC_1_, phaC_2_*, and *phaC_3_* displayed a significant transcriptional upregulation when comparing stationary to late exponential growth phase (Fig. 3D). A slight upregulation was already apparent for *phaC_1_* upon comparing late to early exponential growth phase. In contrast, the expression level of *phaC_Hme_* remained relatively constant over all investigated timepoints. Although less pronounced, a similar trend of upregulation toward the stationary growth phase was observed in the growth-permissive condition (Fig. 3E), which was most pronounced for *phaC_2_*and *phaC*_3_. Simultaneously, *phaC_Hme_* displayed a slight downregulation in later growth stages. Altogether, these observations suggest that the traditionally recognized key enzyme *phaC_Hme_* is at its maximal potential in earlier growth stages, while *phaC_1_, phaC_2_*, and *phaC_3_* show an increased importance in PHA synthesis in later growth phases.

### Effects of valeric acid supplementation on transcriptional expression of *phaC* paralogs

**Figure 4:**
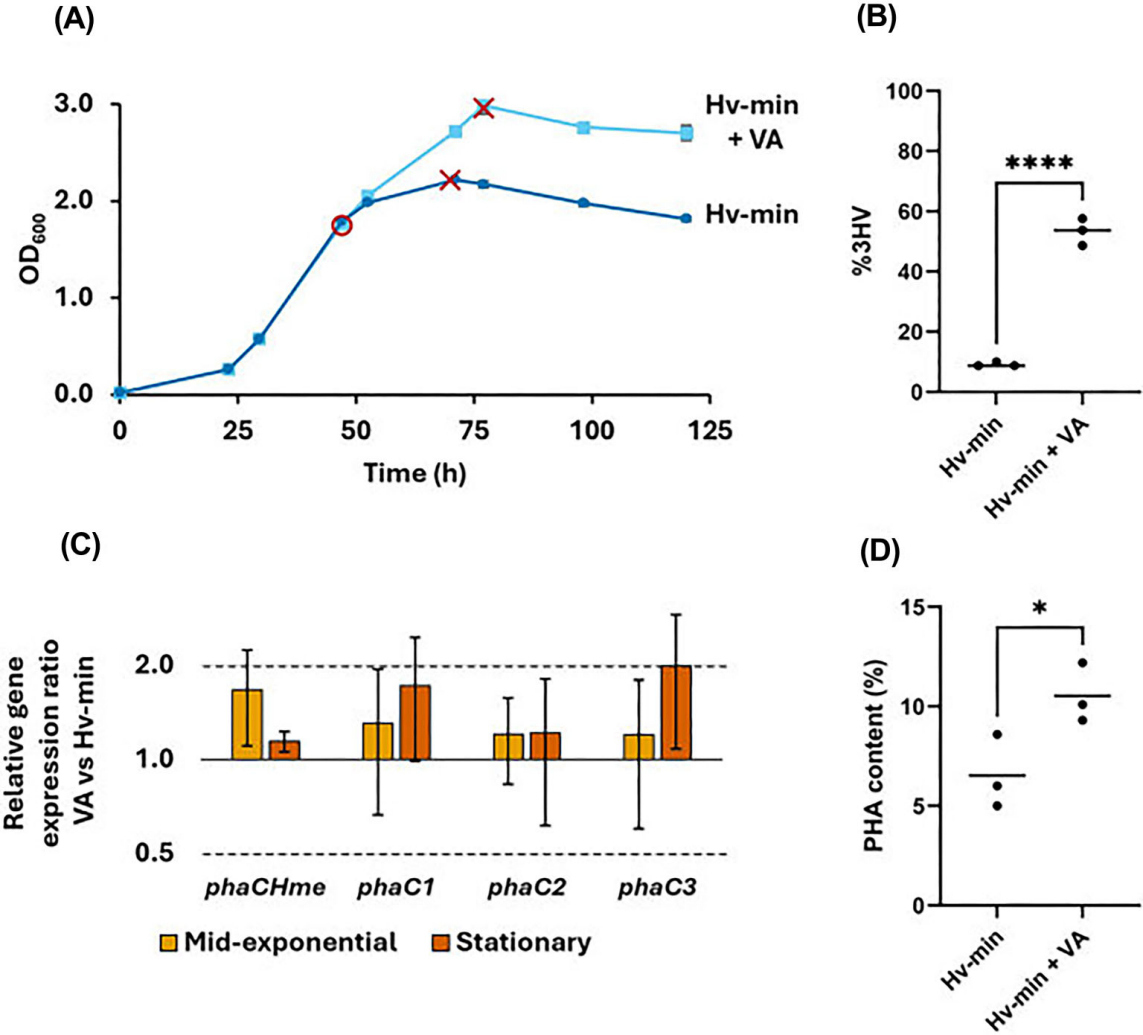
Growth, PHA synthesis and relative gene expression of *phaC* paralogs upon addition of valeric acid. (A) Growth curves of *H. mediterranei* in Hv-min medium with and without valeric acid supplementation, measured as optical density at 600 nm (OD_600_) over time. Values represent the average of three biological replicates; error bars indicate standard deviations. mRNA sampling points are indicated with circles for mid-exponential and crosses for stationary phase. (B) 3-Hydroxyvalerate (%3HV) content in the PHA polymers produced in Hv-min medium with (Hv-min + VA) and without valeric acid (Hv-min), as measured by NMR. Triplicate values were compared using a *t*-test; ^****^ = *P* < .0001. (C) Relative transcriptional expression of *phaC* paralogs in Hv-min medium comparing addition of valeric acid to the absence of it, as determined by RT-qPCR. Values represent the average of three biological replicates and error bars indicate standard deviations. Dotted lines indicate thresholds for 2.0 (upregulation) and 0.5 (downregulation). (D) PHA content in Hv-min medium with and without valeric acid suplementation. Mean values are indicated with a horizontal line. Triplicate values were compared using a *t*-test; * indicates *P* = .05–.01.

Although *H. mediterranei* is inherently capable of producing PHBV (Fig. 3B), previous studies have shown that the 3HV content of the copolymer can be increased by supplementing the medium with a precursor such as valeric acid (Ferre-Guell and Winterburn 2018). Here, we supplemented exponentially growing cells under growth-permissive conditions (Hv-min medium) with 0.5 g l^−1^ valeric acid and monitored its impact on growth and PHA synthesis (Fig. 4). The addition of valeric acid stimulated growth, prolonging the exponential phase and resulting in a higher OD_600_ (2.990 ± 0.090 as compared to 2.220 ± 0.003) (Fig. 4A). The addition of valeric acid significantly increased the 3HV content in the polymer of *H. mediterranei* from 9.2 ± 0.6% to 53.4 ± 3.7% (Fig. 4B; Fig. S9). Moreover, precursor feeding boosted the PHA content from 6.5 ± 1.5% to 10.5 ± 1.2% (Fig. 4D).

Given that PhaC_1_ and PhaC_3_ were previously shown to synthesize PHAs with distinct monomeric compositions upon heterologous expression (Han et al. 2010), we investigated the transcriptional gene expression levels of all *phaC* paralogs in Hv-min medium with and without valeric acid supplementation (Fig. 4C). No significant differential expression was observed in either the mid-exponential or stationary growth phase. This indicates that transcriptional regulation of the paralogs is not responsive to the presence of a 3HV precursor and that it is independent of the copolymer’s 3HV content.

**Figure 5:**
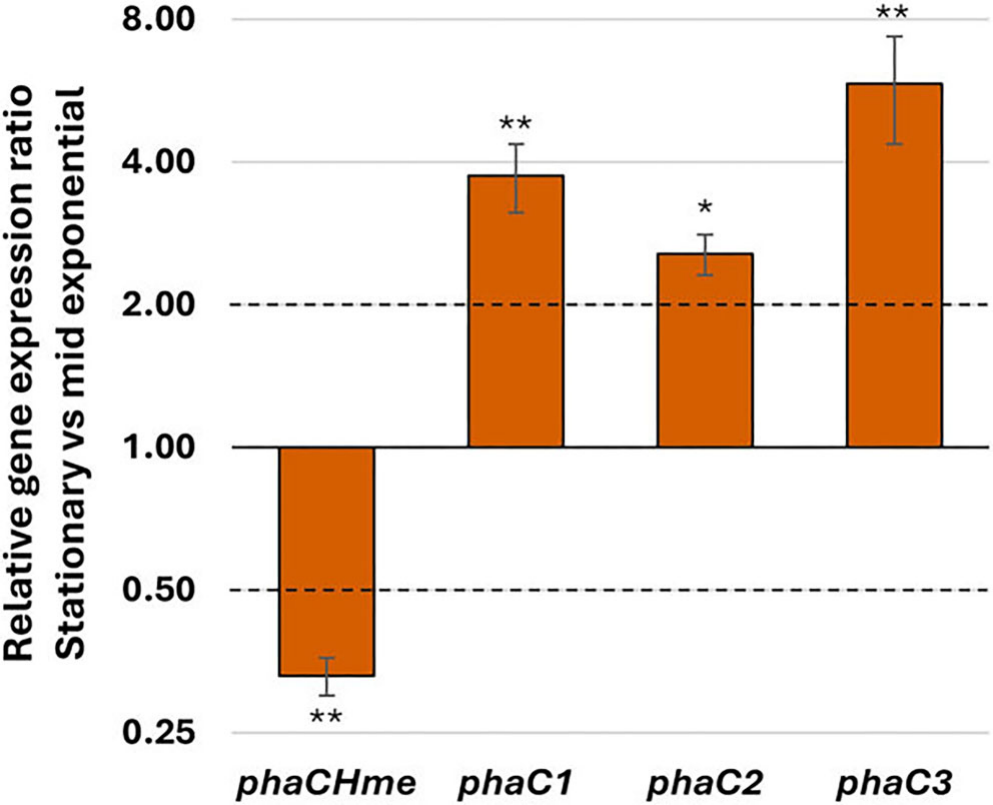
Relative gene expression of *phaC* paralogs in stationary phase relative to mid-exponential phase upon addition of valeric acid. Relative transcriptional expression was determined by RT-qPCR. Ct values were compared using a statistical *t*-test. * indicates *P* = .05–.01, ** indicates *P* = .01–.001. Values represent the average of three biological replicates and error bars indicate standard deviations. Dotted lines indicate thresholds for 2.0 (upregulation) and 0.5 (downregulation).

The growth phase dependence of gene expression of the *phaC* paralogs was also investigated in precursor-supplemented medium by comparing the stationary and mid-exponential growth phase. The three paralogs *phaC1, phaC2*, and *phaC3* again showed significant upregulation in the stationary phase (relative expression ratios of 3.75 ± 0.62, 2.56 ± 0.25, and 5.86 ± 1.50 for *phaC1, phaC2*, and *phaC3*, respectively) while the main subunit *phaC_Hme_* showed a downregulation (relative gene expression ratio of 0.33) (Fig. 5). These results further corroborate an increased importance of the *phaC1, phaC2*, and *phaC3* paralogs in later growth stages.

### Differences in relative transcription levels of the *phaC* paralogs

**Figure 6:**
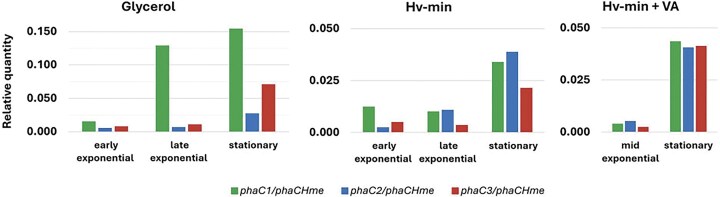
Relative quantity of transcript levels of the main *phaC* subunit (*phaC_Hme_*) compared to each paralog in all investigated conditions. Values for each gene are derived by relating measured Ct values to their respective standard curves.

Given that all paralogs are actively transcribed under the investigated conditions, we next set out to analyse potential differences in relative transcription levels of the *phaC_1_, phaC_2_*, and *phaC_3_* paralogs compared to the main *phaC_Hme_* paralog in different growth phase stages (Fig. 6). Using standard curves generated with gDNA as a template, measured Ct values were converted into input DNA quantities, which were then used as an approximation of transcript levels. Throughout the growth process, *phaC_Hme_* was transcribed at significantly higher levels than the three other paralogs (*phaC1, phaC2*, and *phaC3*) across all investigated media (Fig. 6). However, the difference in transcription levels decreased as growth progressed, with the gap between the main subunit and the other paralogs becoming less pronounced in the late exponential and stationary phases.

## Discussion

Our work has shown that *H. mediterranei* is the only species within the class *Halobacteriales* that encodes four distinct PhaC paralogs. Structural conservation across the catalytic cores of all four enzymes, combined with variations in the terminal regions, indicates a shared enzymatic ancestry with potentially divergent functional capacities. The consistent presence of a conserved N-terminal α-helix in all paralogs further suggests that interaction with PhaE remains possible. A screening of 225 *Halobacteriales* genome sequences revealed that ~17% are also predicted to encode multiple paralogs, though only two or three and predominantly PhaC_1_-like or PhaC_3_-like homologs. No other species was found to encode a homolog of PhaC_2_, which was previously shown to be functionally inactive (Han et al. 2010). This suggests that PhaC_2_ is unique to *H. mediterranei*.

Whereas a previous study reported that only *phaC_Hme_* was actively transcribed and that the three other paralogs represented cryptic genes (Han et al. 2010), our results demonstrate that all *phaC* paralogs are expressed under different PHA-producing growth conditions. In line with previous findings (Han et al. 2010, Lu et al. 2008), our results confirm the role of PhaC_Hme_ as the primary PhaC subunit in *H. mediterranei*, as it consistently exhibited the highest transcription levels. This conclusion is further supported by its genomic context, with *phaC_Hme_* being colocated with *phaE_Hme_* in a single operon, both being under the regulation of transcription factor PhaR (Lu et al. 2008). When looking beyond *Halobacteriales*, the occurrence of multiple PhaC paralogs is not unusual. In *Rhodospirillum rubrum*, several paralogs (PhaC_1_ and PhaC_3_) are functionally redundant and contribute little to PHA metabolism under standard growth conditions (Jin and Nikolau 2012). In *Bradyrhizobium japonicum*, five PhaC paralogs are encoded in the genome of which only two (PhaC1 and PhaC2) are significantly expressed (Quelas et al. 2013). Although both are expressed at similar levels in wild-type cells, no PHA accumulation was observed in a ΔPhaC1 strain, whereas PHA production was increased in a ΔPhaC2 strain, indicating a complex interplay between the two enzymes. In the same study, it was hypothesized that the paralogs form a heterodimer and mutually balance out each other’s activity (Quelas et al. 2013).

As previous reports suggested that multiple PhaC paralogs could be involved in the synthesis of polymers with distinct monomeric compositions (Han et al. 2010, Chen et al. 2020), we assessed the effects of precursor addition on the transcriptional expression of the *phaC* genes in *H. mediterranei*. PHBV produced by this species typically contains about 10% 3HV, independent of the carbon source (Koller et al. 2007a, Chen et al. 2006, Bhattacharyya et al. 2012, 2014), consistent with our findings on glycerol and Hv-min media. The potential of valerate as a 3HV precursor in *H. mediterranei* had already been investigated previously (Koller et al. 2007b, Han et al. 2015); however, our approach resulted in considerably higher 3HV incorporation at the tested precursor concentration compared to earlier reports [21.8% for Koller et al. (2007b) at 8 mM valerate; 20.8% for Han et al. (2015) at 6.5 mM valerate; 53.4% in our study at 4.9 mM valerate]. A possible explanation for this discrepancy is our use of valeric acid as the source of valerate, which might cause pH stress, thereby altering PHA production characteristics. Despite the large difference in 3HV content, we observed that the regulation of all *phaC* paralogs appears to be independent of PHA content or composition.

Although the relative expression levels of *phaC_1_, phaC_2_*, and *phaC_3_*were markedly lower than that of the canonical *phaC_Hme_*, they were nonetheless regulated, indicating potential physiological relevance. This regulation was growth-phase-dependent, with *phaC_1_* and *phaC_3_* being significantly upregulated during the stationary phase, while expression of the main subunit remained constant or showed a slight downregulation, thereby gradually lowering the difference between the expression level of *phaC_Hme_* and those of *phaC_1_, phaC_2_*, and *phaC_3_*. It is possible that transcripts of *phaC_1_, phaC_2_*, and *phaC_3_* went undetected in the previous study due to transcription being assessed exclusively during the exponential growth phase (Han et al. 2010). However, the detection of transcription across all paralogs, with consistently higher levels of *phaC_Hme_*compared to *phaC_1_, phaC_2_*, and *phaC_3_* is consistent with values previously reported in an RNA-sequencing study, also in exponential growth phase (Chen et al. 2019, 2020).

Interestingly, in other PHA-producing *Halobacteriales*, different paralog classes—except for true PhaC_2_-like variants—can be found within *phaEC*-like operons, corroborating the hypothesis that the functional relevance of the paralogs in *H. mediterranei* is influenced by regulated expression. The tight regulation of the noncanonical *phaC* paralogs suggests the existence of a broader regulatory network involving multiple *trans*-acting factors. This includes the phosphoenolpyruvate synthase-like protein PspR, whose gene is encoded in a divergent operon with *phaC_1_* (Fig. 1A). Deletion of *pspR* has been shown to correlate with higher transcriptional expression of *phaC_1_* and *phaC_3_*, although the underlying mechanisms are not entirely clear (Chen et al. 2020, 2024). Additional regulators might include PhaR and Fnr-like proteins (Mitra et al. 2021, Chen et al. 2024), and it can be hypothesized that the expression of the paralogs is also subject to posttranscriptional regulation. In this context, it is noteworthy that the *phaC_1_* paralog possesses an exceptionally long 5′-UTR region (∼350 nt) (Fig. S10) (Martinez Pastor et al. 2025). Future research could focus on unraveling the underlying regulatory network, with the hypothesis that the noncanonical *phaC* paralogs may be upregulated under diverse stress conditions, not only stationary-phase growth, and could contribute to modulating the properties of the synthesized biopolymer.

## Supplementary Material

fnag006_Supplemental_File
