# Supplementary material for: Insights into transcriptional expression and putative functions of multiple polyhydroxyalkanoate synthase paralogs in Haloferax mediterranei
Source: FEMS Microbiol Lett. 2026 Jan 19;373:fnag006. doi: 10.1093/femsle/fnag006 (PMC12863089; doi:10.1093/femsle/fnag006)
Supplement: fnag006_Supplemental_File [file fnag006_supplemental_file.pdf]

SUPPLEMENTARY MATERIALS - Insights into transcriptional expression and putative functions of multiple *phaC* paralogs in *Haloferax mediterranei*

Supplementary Note S1: Relative gene expression ratios (R, Equation 2) were calculated according to the Pfaffl method. Inputs to the Pfaffl equation are modified primer efficiencies (E, Equation 1) and Ct values of both the target genes and a housekeeping gene under a sample and a control condition. Both the *ffs* and *tbp* genes were investigated as housekeeping genes in all media. As the Pfaffl method allows to take the exact amplification efficiencies into account in the calculation of gene expression ratios, stability of the housekeeping gene rather than its amplification efficiency was used as the main criterion for choice of the housekeeping gene. For the analysis over different growth stages in the glycerol and the Hv-min+VA media, *tbp* was used as the stable housekeeping gene whereas *ffs* was used in Hv-min medium. For comparison of the Hv-min and Hv-min+VA conditions, the geometric mean of both *tbp* and *ffs* was used according to Equation 3. All input Ct values were the average of two technical replicates and three biological replicates. The stability of the candidate housekeeping genes is depicted in **Supplementary Figure S1**.

$$E = 1 + \left[\frac{amplification\ efficiency\ (\%)}{100}\right] \tag{1}$$

$$R = \frac{{E_{target}}^{\Delta Ct\ (control-sample)}}{{E_{HK}}^{\Delta Ct\ (control-sample)}} \tag{2}$$

$$R = \frac{{E_{target}}^{\Delta Ct\ (control-sample)}}{GeoMean\left[{E_{HK}}^{\Delta Ct\ (control-sample)}\right]} = \frac{{E_{target}}^{\Delta Ct\ (control-sample)}}{\prod_{i=1}^{n}\left[{E_{HK}}^{\Delta Ct\ (control-sample)}\right]_i^{\frac{1}{n}}} \tag{3}$$

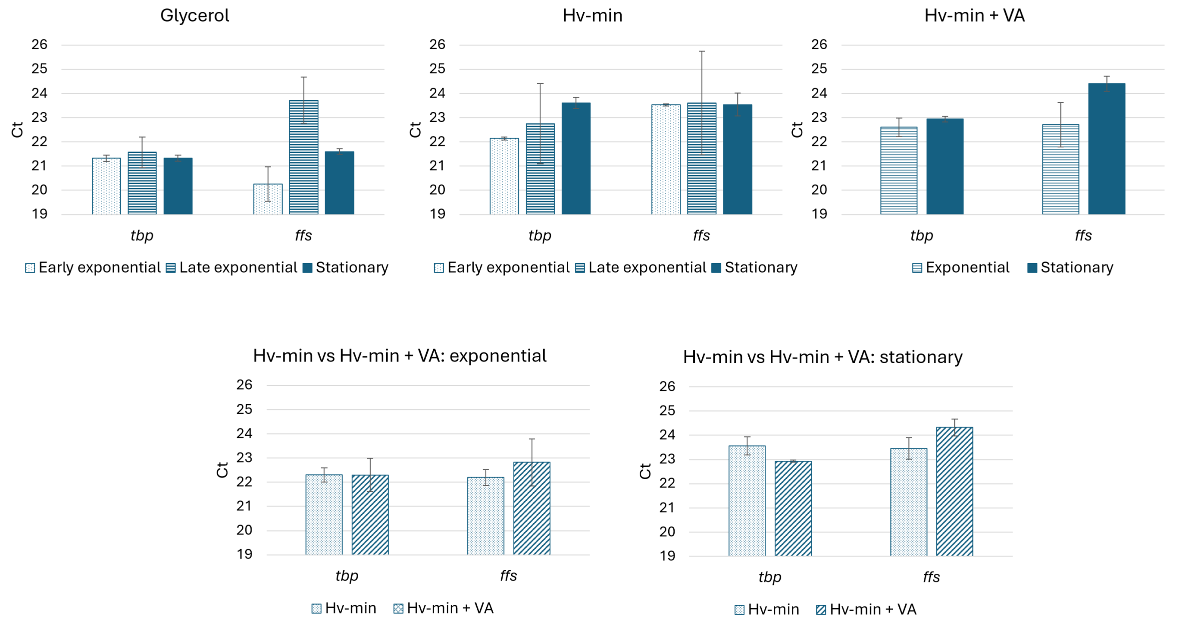

Supplementary Figure S1: Stability of candidate housekeeping genes *tbp* and *ffs* across the different experimental set-ups.

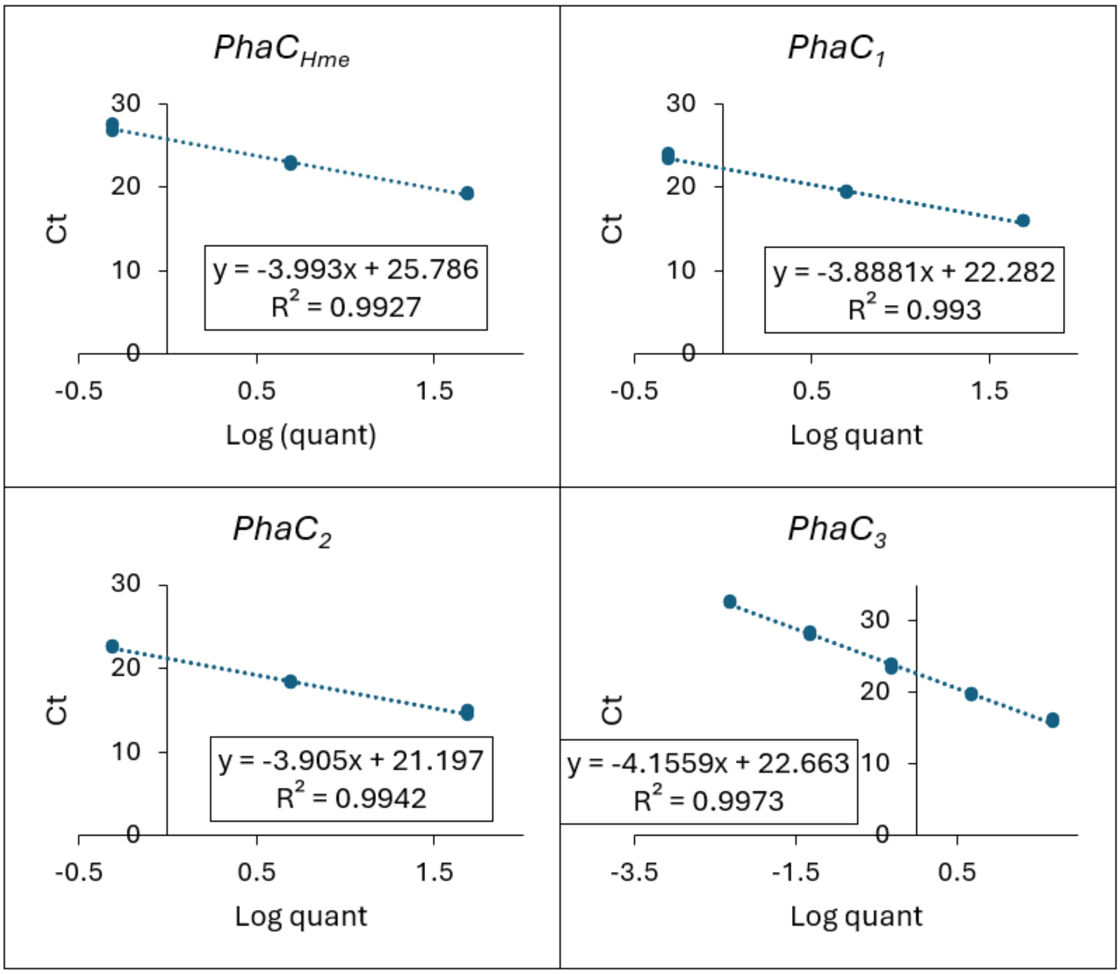

Supplementary Figure S2: Standard curves relating measured Ct values to input DNA quantities for all paralogs, measured on gDNA. The equations of the standard curves are used for estimation of the transcript levels.

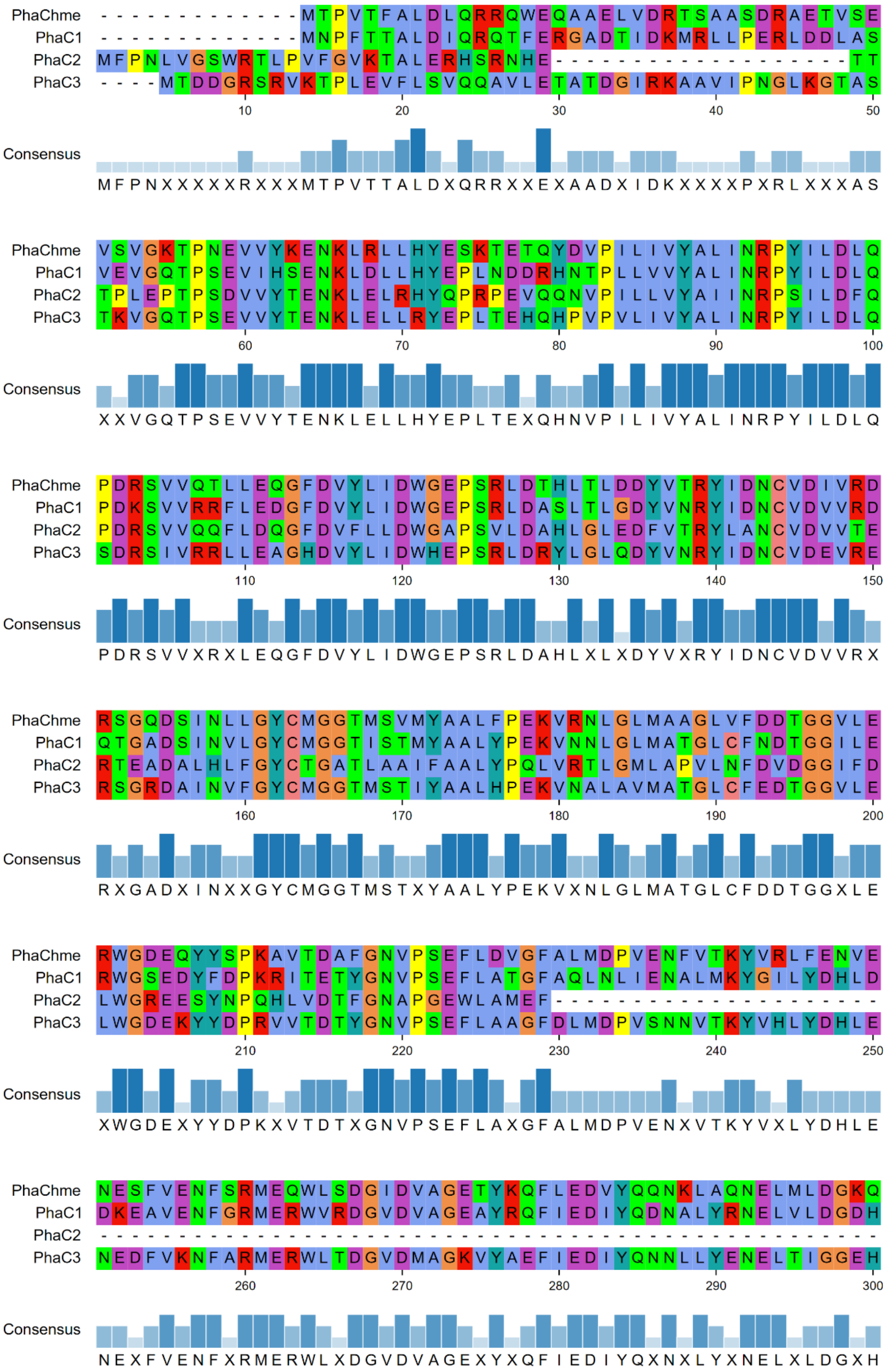

Supplementary Figure S3: Multiple sequence alignment of the four PhaC paralogs from *Haloferax mediterranei*. The alignment was visualized using pymsaviz with the Clustal color scheme. Consensus residues are indicated at the bottom of each panel. For clarity, the alignment is presented in two consecutive parts (1/2)

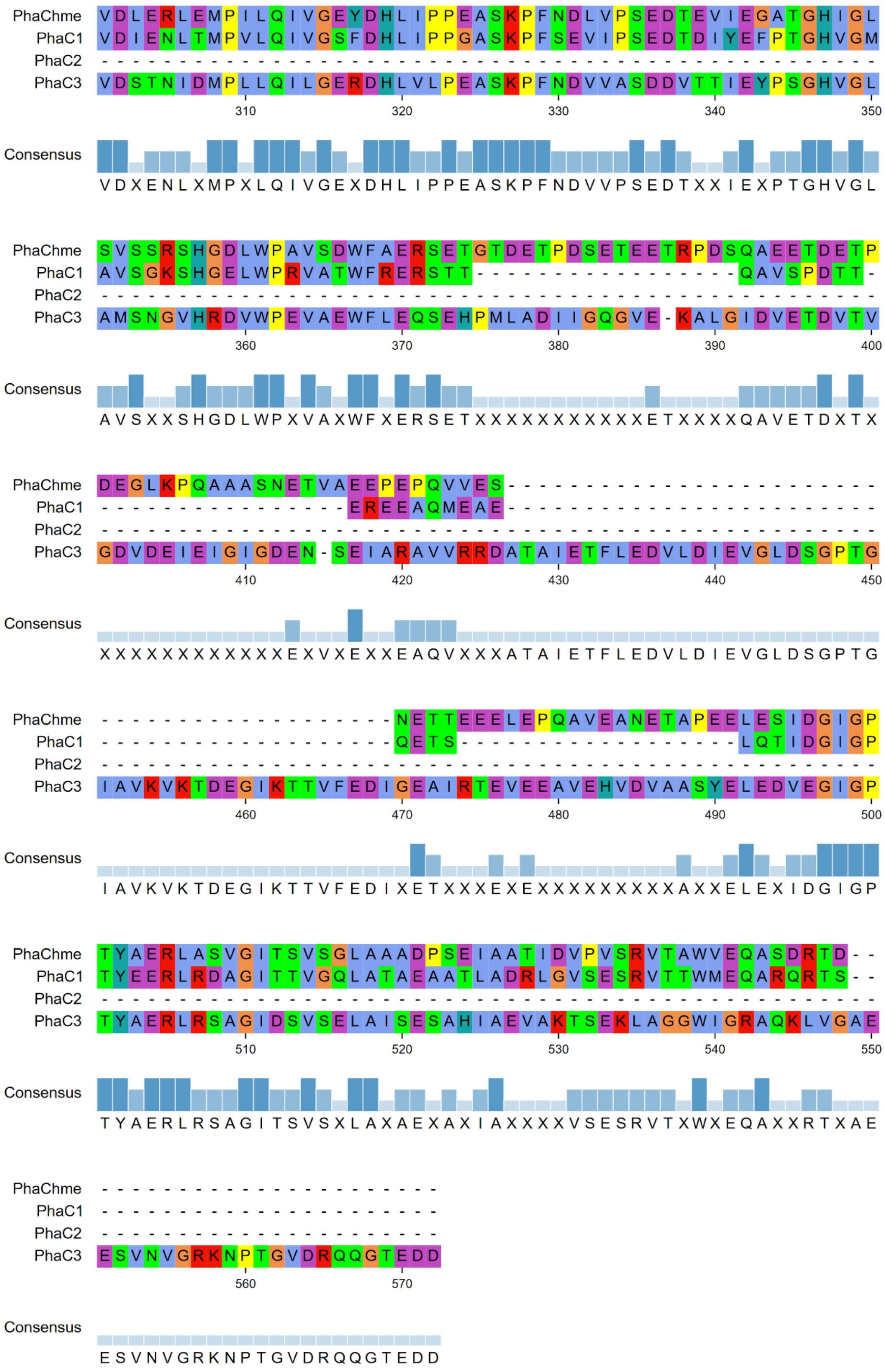

Supplementary Figure S3: Multiple sequence alignment of the four PhaC paralogs from *Haloferax mediterranei*. The alignment was visualized using pymsaviz with the Clustal color scheme. Consensus residues are indicated at the bottom of each panel. For clarity, the alignment is presented in two consecutive parts (2/2).

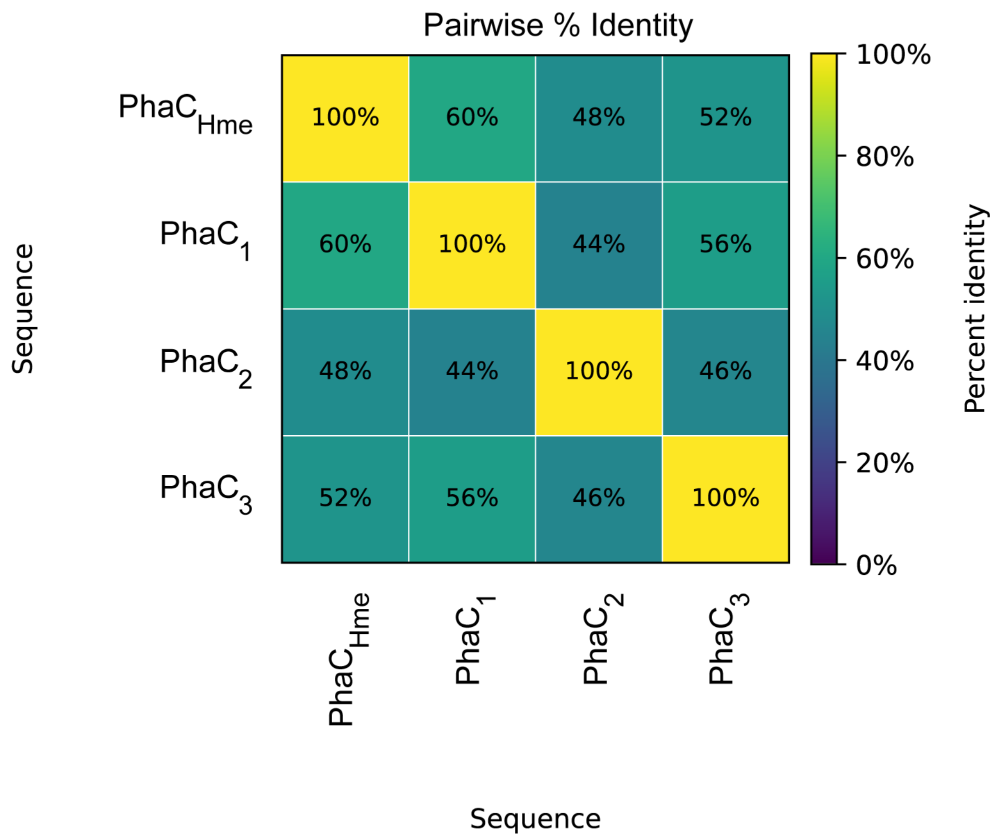

Supplementary Figure S4: Pairwise percentage identity matrix of aligned PhaC paralog sequences. Values represent the percentage of identical residues between each pair of sequences, calculated from the multiple sequence alignment with pairwise deletion of positions containing gaps.

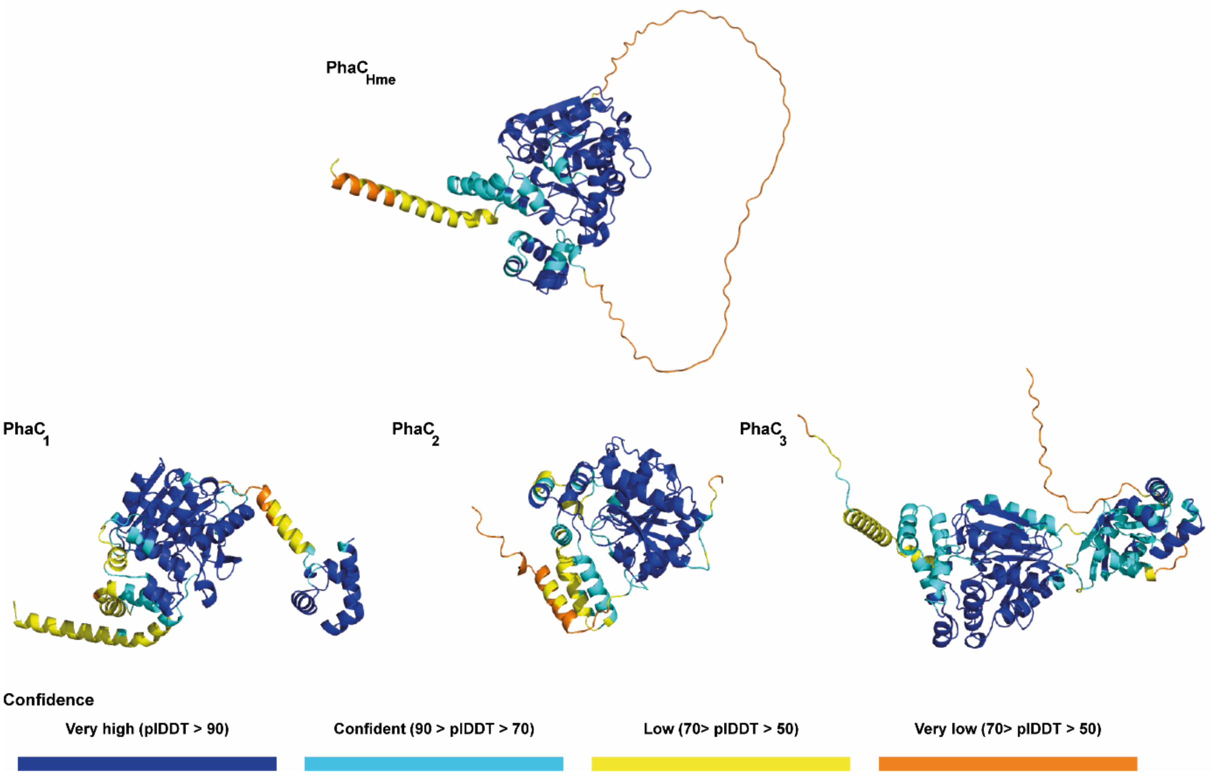

Supplementary Figure S5: AlphaFold models of $PhaC_{Hme}$ and its three paralogs. The structures are coloured based on their confidence scores according to the legend below.

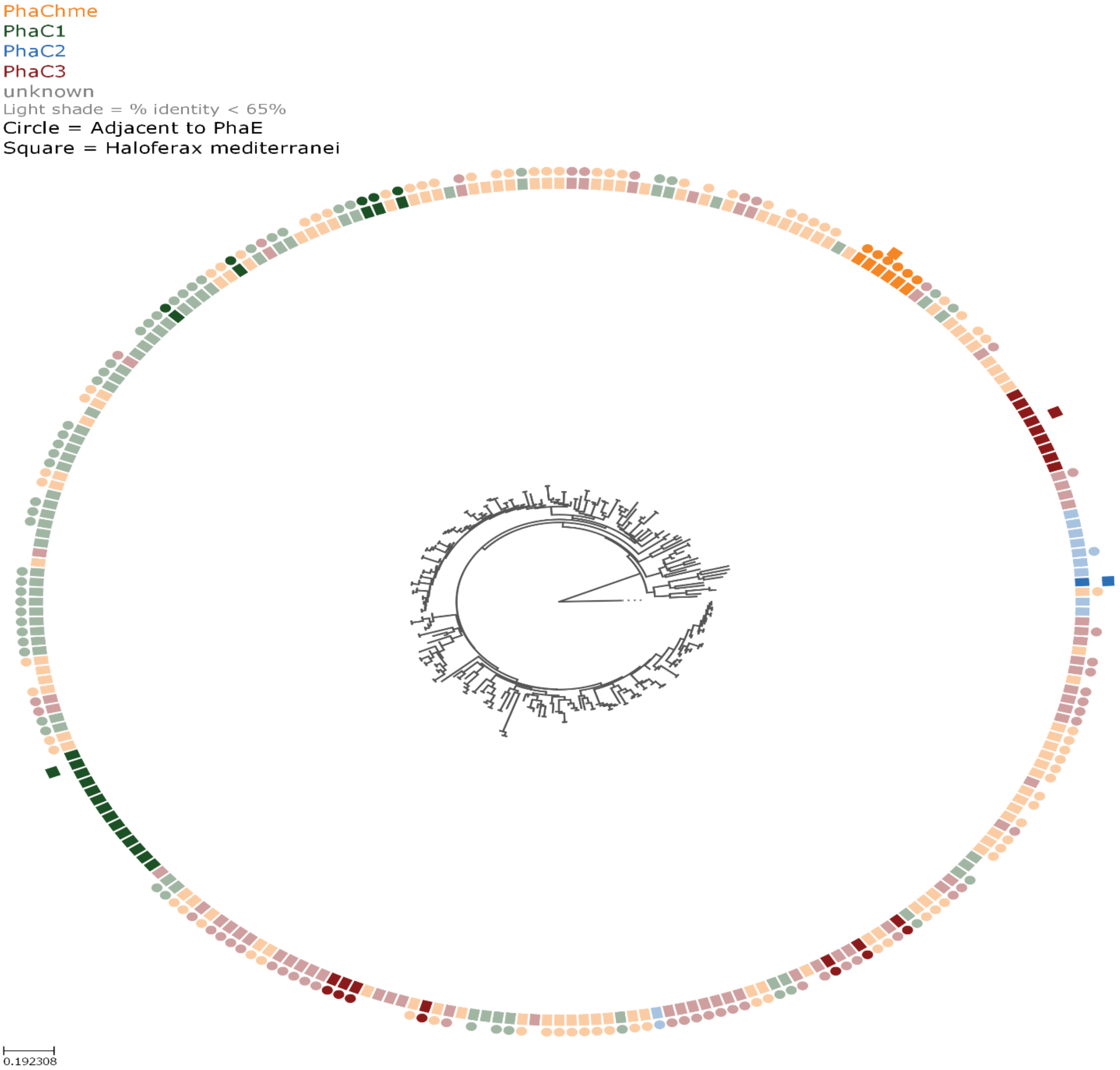

Supplementary Figure S6: Circular phylogram of PhaC paralogs with class annotation and genomic context markers. A Newick tree of PhaC sequences is shown in circular layout with branch lengths preserved. Leaf labels are hidden for clarity. For each leaf, a short radial tick encodes the assigned PhaC class: **PhaChme** (orange), **PhaC1** (dark green), **PhaC2** (blue), **PhaC3** (maroon); **unknown** is grey. Tick shading reflects the class score (percent sequence identity): **lightened** ticks indicate identity < **65%**, full color ≥ **65%**. Two markers report genomic context: a **filled circle** denotes that the PhaC locus is **adjacent to phaE**, and a **filled square** marks **Haloferax mediterranei** sequences. Anti-overlap sizing was applied so ticks and markers scale to leaf density while preserving branch lengths. Figure rendered with ETE3.

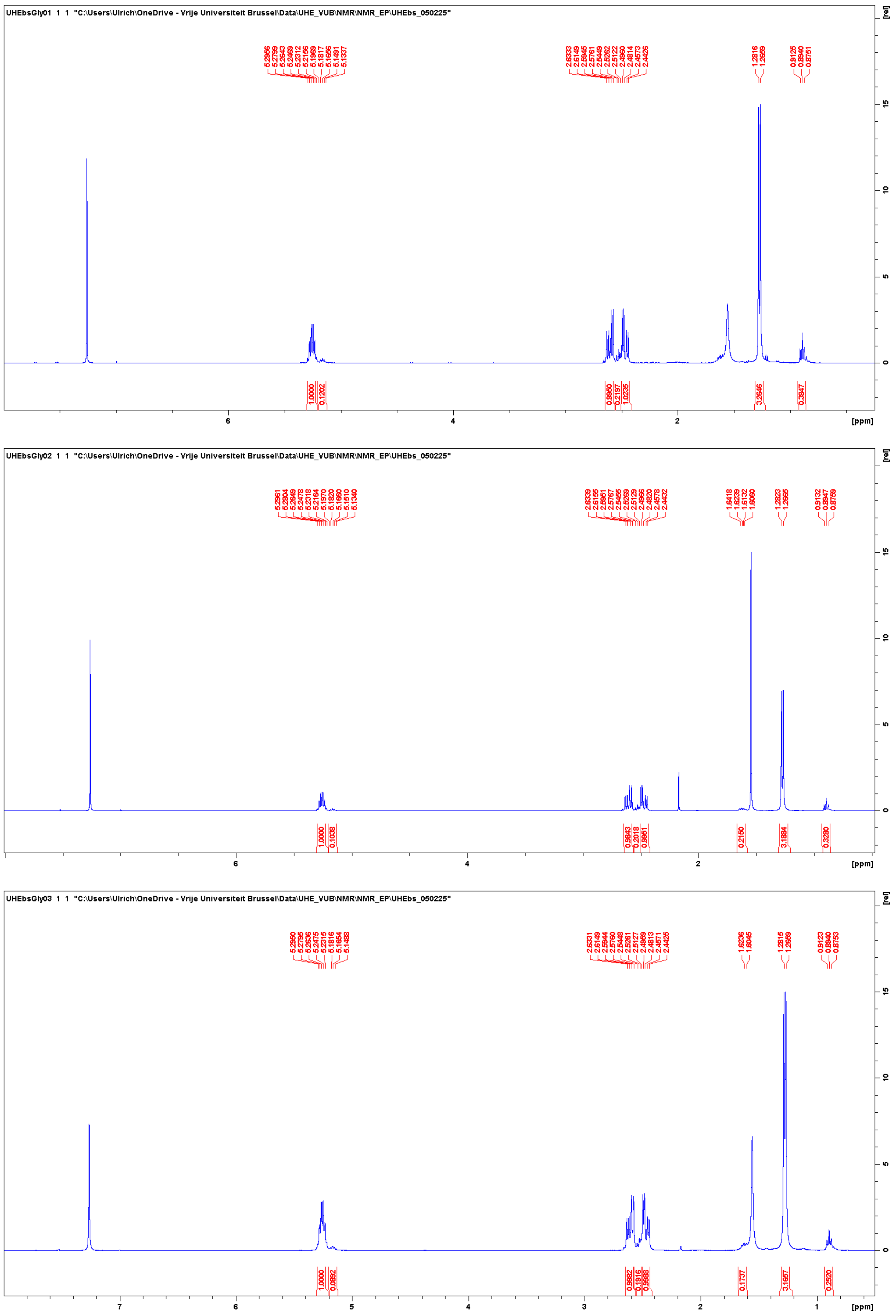

Supplementary Figure S7: ^{1}H-NMR spectra of PHBV polymers extracted in triplicate from *Haloferax mediterranei* cells grown on the glycerol minimal medium.

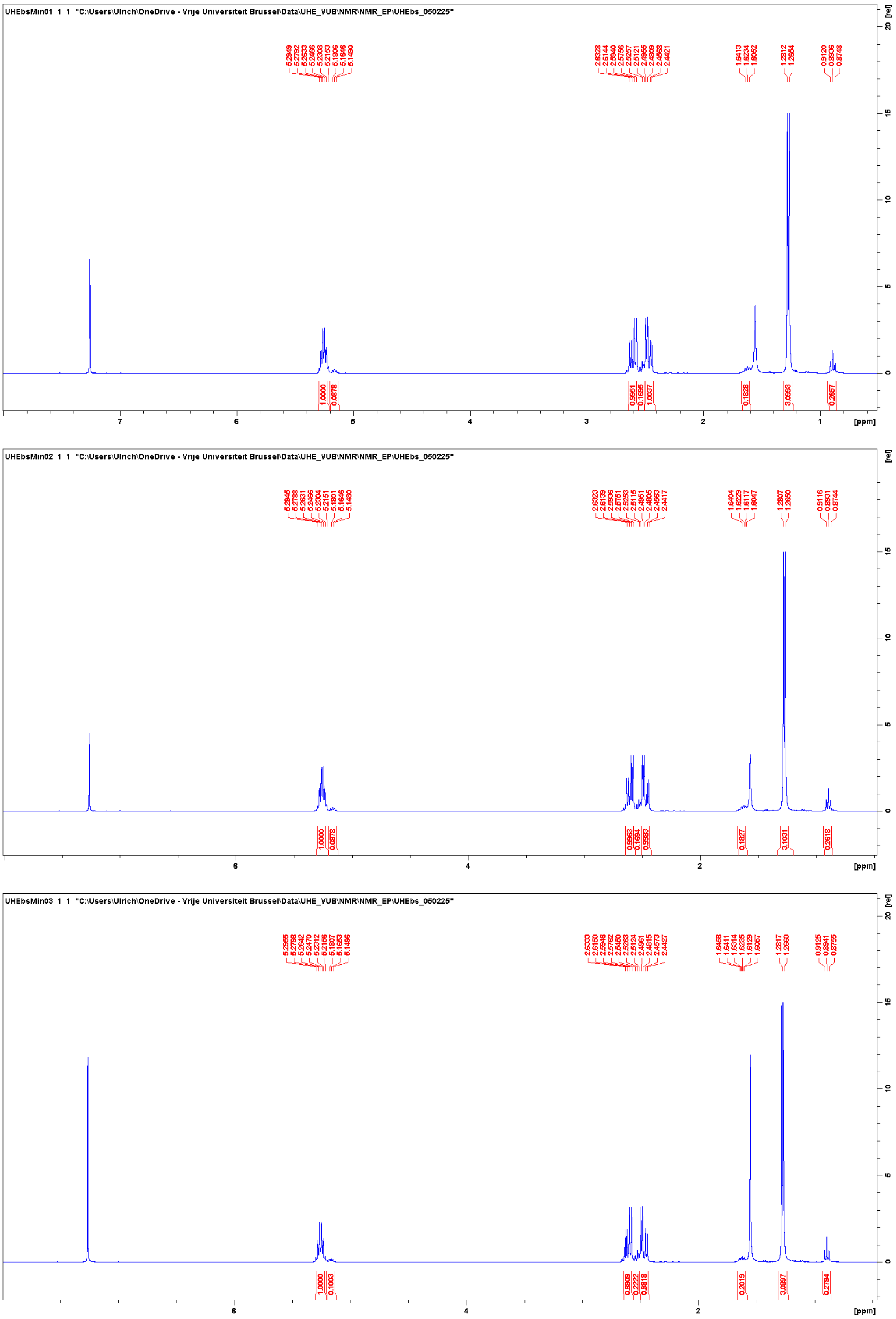

Supplementary Figure S8: ^{1}H-NMR spectra of PHBV polymers extracted in triplicate from *Haloferax mediterranei* cells grown on the Hv-min minimal medium.

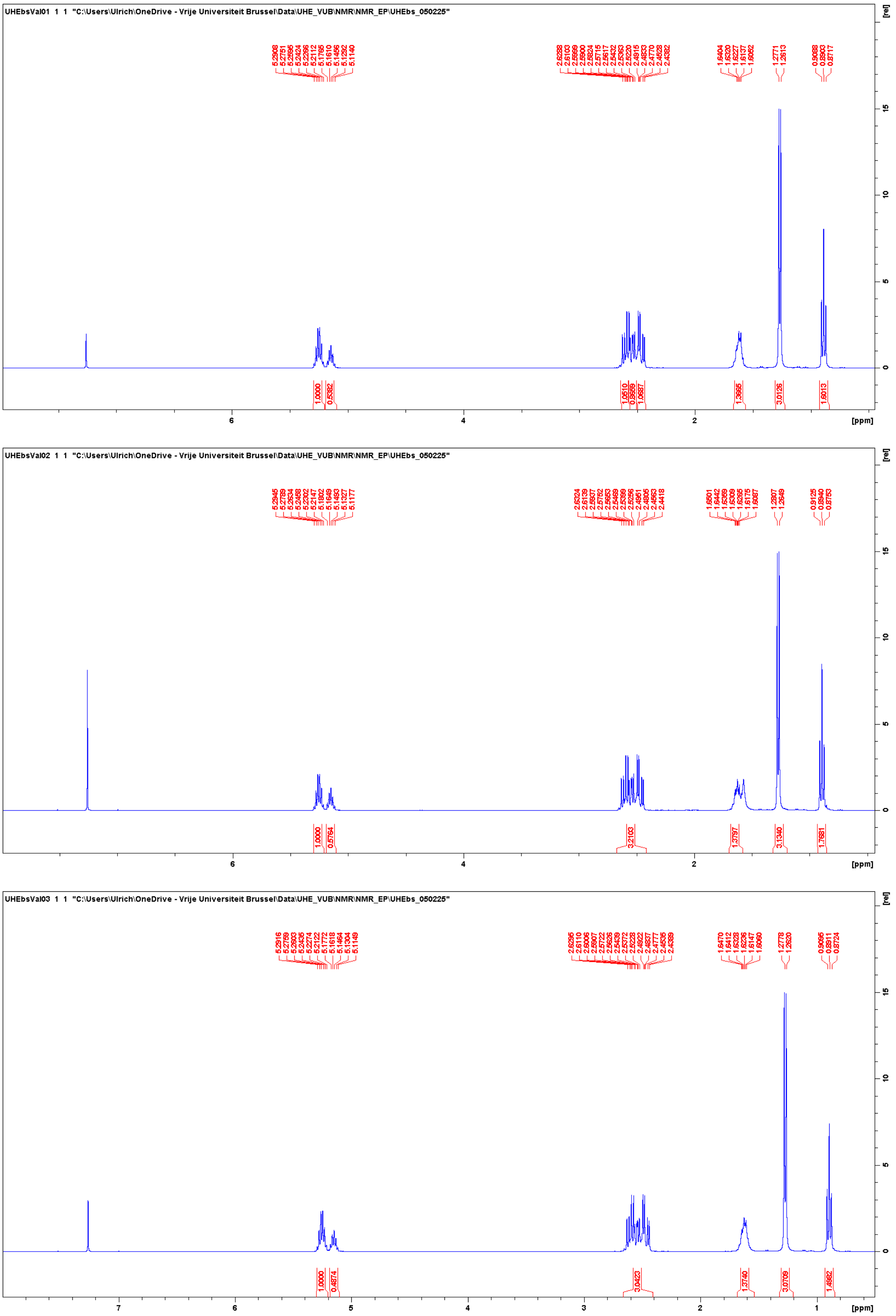

Supplementary Figure S9: ^{1}H-NMR spectra of PHBV polymers extracted in triplicate from *Haloferax mediterranei* cells grown on the Hv-min minimal medium with the addition of valeric acid.

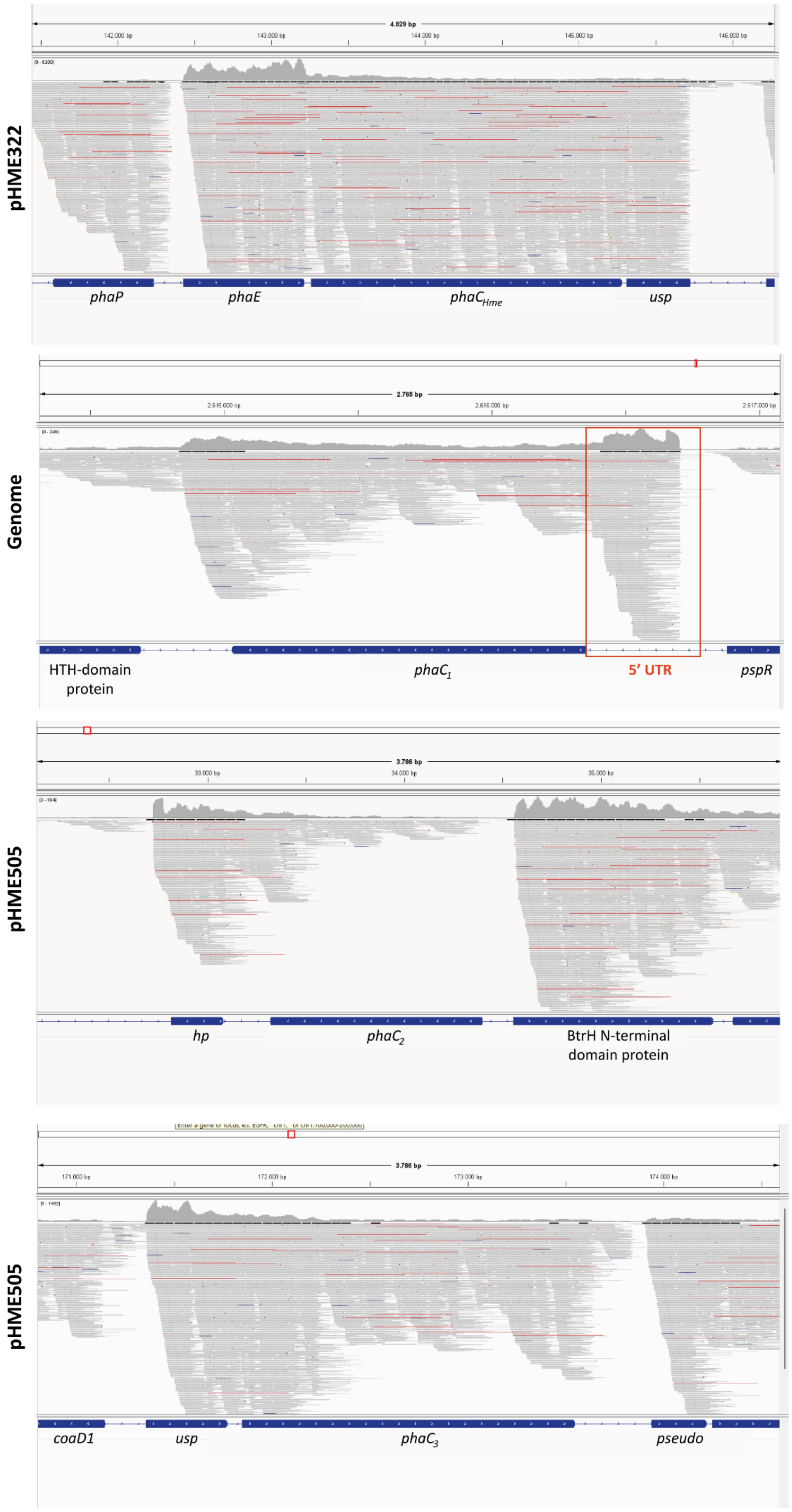

Supplementary Figure S10: Visualization of the mapped RNA-seq data published (Run: **SRR30916285**) previously by Martinez-Pastor *et al*. (2025) zoomed in on the genomic location of the paralogs. In red, the location of a hypothetical 5’UTR is boxed downstream of the *phaC*$_1$ gene.

Supplementary Table S1: List of the genes monitored by RT-qPCR with their respective primers (forward F and reverse R) sequences and amplification efficiencies.

Gene name (locus)	Gene abbreviation	F/R	Sequence (5' → 3')	Amplification efficiency (%)
TATA-box-binding protein (E6P09_03900)	*tbp*	F	ATCTCAACGCCATCGCAATCG	80.2
		R	TACCCGACCCGAAGAGAAGTGC	
Signal recognition particle RNA (E6P09_08165)	*ffs*	F	AGTTAGGCCCTGCTCTTCACC	68.1
		R	GGTTTCTACGTTGGCTTCCG	
PHA synthase subunit PhaC (E6P09_17995)	$phaC_{Hme}$	F	GACTACGTGACTCGGTACATCG	78.0
		R	CAGTACCCGAGAAGGTTAATCG	
PHA synthase subunit PhaC1 (E6P09_13380)	$phaC_1$	F	CAACAATCTCGGACTCATGG	80.8
		R	TCCGTACGTTTCGGTAATCC	
PHA synthase subunit PhaC2 (E6P09_15240)	$phaC_2$	F	CCGCTATCTCAGCCTCTACG	80.3
		R	TGTCAAAACCCCACTGAAGC	
PHA synthase subunit PhaC3 (E6P09_15825)	$phaC_3$	F	CTCGCTATCTCCGAAAGTGC	74.0
		R	TGGGGTTCTTTCTACCAACG	

Supplementary Table S2: Overview of equations of standard curves of all paralogs, used for estimation of transcript levels.

Gene abbreviation	Equation of standard curve
$phaC_{Hme}$	Ct = -3.993 · log(quant) + 25.786
$phaC_1$	Ct = -3.888 · log(quant) + 22.282
$phaC_2$	Ct = -3.905 · log(quant) + 21.197
$phaC_3$	Ct = -4.156 · log(quant) + 22.663
